# Guideline-Based Cardiovascular Risk Assessment Delivered by an mHealth App: Development Study

**DOI:** 10.2196/50813

**Published:** 2023-12-08

**Authors:** Fabian Starnecker, Lara Marie Reimer, Leon Nissen, Marko Jovanović, Maximilian Kapsecker, Susanne Rospleszcz, Moritz von Scheidt, Johannes Krefting, Nils Krüger, Benedikt Perl, Jens Wiehler, Ruoyu Sun, Stephan Jonas, Heribert Schunkert

**Affiliations:** 1 Department of Cardiology German Heart Center Munich Technical University of Munich Munich Germany; 2 Partner Site Munich Heart Alliance German Center for Cardiovascular Disease (Deutsches Zentrum für Herz-Kreislauf-Forschung eV) Munich Germany; 3 School for Computation, Information and Technology Technical University of Munich Munich Germany; 4 Institute for Digital Medicine University Hospital Bonn Bonn Germany; 5 Institute of Epidemiology Helmholtz Zentrum München German Research Center for Environmental Health (GmbH) Neuherberg Germany; 6 Chair of Epidemiology Institute for Medical Information Processing, Biometry, and Epidemiology, Faculty of Medicine Ludwig Maximilian University of Munich Munich Germany; 7 Department of Sport and Health Sciences Technical University of Munich Munich Germany; 8 BioM Biotech Cluster Development GmbH - BioM Munich Germany; 9 Medical Graduate Center Technical University of Munich Munich Germany

**Keywords:** cardiovascular disease, cardiovascular risk assessment, HerzFit, mobile health app, mHealth app, public information campaigns, prevention, risk calculator, mobile phone

## Abstract

**Background:**

Identifying high-risk individuals is crucial for preventing cardiovascular diseases (CVDs). Currently, risk assessment is mostly performed by physicians. Mobile health apps could help decouple the determination of risk from medical resources by allowing unrestricted self-assessment. The respective test results need to be interpretable for laypersons.

**Objective:**

Together with a patient organization, we aimed to design a digital risk calculator that allows people to individually assess and optimize their CVD risk. The risk calculator was integrated into the mobile health app HerzFit, which provides the respective background information.

**Methods:**

To cover a broad spectrum of individuals for both primary and secondary prevention, we integrated the respective scores (Framingham 10-year CVD, Systematic Coronary Risk Evaluation 2, Systematic Coronary Risk Evaluation 2 in Older Persons, and Secondary Manifestations Of Arterial Disease) into a single risk calculator that was recalibrated for the German population. In primary prevention, an individual’s heart age is estimated, which gives the user an easy-to-understand metric for assessing cardiac health. For secondary prevention, the risk of recurrence was assessed. In addition, a comparison of expected to mean and optimal risk levels was determined. The risk calculator is available free of charge. Data safety is ensured by processing the data locally on the users’ smartphones.

**Results:**

Offering a risk calculator to the general population requires the use of multiple instruments, as each provides only a limited spectrum in terms of age and risk distribution. The integration of 4 internationally recommended scores allows risk calculation in individuals aged 30 to 90 years with and without CVD. Such integration requires recalibration and harmonization to provide consistent and plausible estimates. In the first 14 months after the launch, the HerzFit calculator was downloaded more than 96,000 times, indicating great demand. Public information campaigns proved effective in publicizing the risk calculator and contributed significantly to download numbers.

**Conclusions:**

The HerzFit calculator provides CVD risk assessment for the general population. The public demonstrated great demand for such a risk calculator as it was downloaded up to 10,000 times per month, depending on campaigns creating awareness for the instrument.

## Introduction

Cardiovascular disease (CVD) risk is mostly driven by modifiable risk factors such as hypercholesterolemia, arterial hypertension, diabetes mellitus (DM), and smoking. An unhealthy lifestyle favors many of these risk factors and is therefore deemed to play a major role in CVD. Thus, the promotion of a healthy lifestyle and the effective treatment of modifiable risk factors are essential for CVD prevention [1]. In reality, however, many people are unaware of opportunities to improve their health and life expectancy. Identifying these individuals at risk and ensuring CVD prevention remains challenging. Systematic screening programs might offer a solution for this problem. However, such programs tend to be expensive and have inconsistent clinical outcomes [2,3]. Self-identification of modifiable risk factors could increase the awareness of health risks. Digital applications, including mobile health (mHealth) apps, could play a role in this regard by providing risk assessment software. In recent years, web-based cardiovascular risk calculators demonstrated great public interest [4]. Clinical validity and understandability have been shown to be critical for the usefulness of such tools. Most of the available web-based calculators base their prediction on well-validated 10-year CVD risk scores [5]. As absolute risk percentages have been found to be difficult to understand for laypersons, calculators presenting a heart age instead have become increasingly popular worldwide [6-9]. These calculators compare the individual risk level to the average risk level. The individual heart age matches the age of a person with the same predicted risk but with average levels of modifiable risk factors [10,11]. If the cardiovascular risk is increased, the estimated heart age exceeds the chronological age. The potential loss of life years is intended to motivate users to better control their risk factors and to adhere to a healthier lifestyle [9]. Simple risk scores such as the Framingham Risk Score (FRS) for 10-year risk of CVD have been shown to be capable of identifying at-risk individuals and can be used for such self-assessment tools [5,12]. As the FRS covers risk only for primary prevention in an age spectrum from 30 to 74 years, other scores are needed to cover a wider age range as well as individuals with previous CVD events. Finally, these scores come from different regions such that the application in a specific region requires recalibration. Here, we report the development of a risk calculator for self-assessment recalibrated to the German population and usable for a wide range of age groups in both primary and secondary prevention.

## Methods

### Overview

The HerzFit risk calculator was developed together with the Deutsche Herzstiftung eV (German Heart Foundation), which is the largest nonprofit and independent patient advocacy group in the field of heart disease in Europe (>100,000 members). The scientific basis of the risk calculator, including the selection of risk scores, was determined together with the scientific advisory board of the German Heart Foundation. Ideas for the practical implementation of the risk calculator, including its communication strategy, were developed together with representatives of the potential target group. These were invited to an interdisciplinary workshop together with our team of physicians from the German Heart Center Munich, medical informaticians from the Technical University of Munich, representatives of the German Heart Foundation, and app designers.

### CVD Risk Assessment

The HerzFit risk calculator provides CVD risk assessment for both primary and secondary prevention. For people without known atherosclerotic disease, the European Society of Cardiology Systematic Coronary Risk Evaluation 2 (SCORE2; applicable to individuals aged 40-69 years) and SCORE2–Older Persons (SCORE2-OP; applicable to individuals aged 70-89 years) models for moderate-risk regions were used. The SCORE2 models base their age-specific, sex-specific, and risk region–specific estimations on systolic blood pressure (SBP), smoking status, and cholesterol levels. The SCORE2-OP version additionally considers DM as a predictor [13,14]. For younger people (aged <40 years), a recalibrated version of the original FRS for 10-year risk of CVD was applied. In addition to age and sex, the FRS uses SBP, antihypertensive treatment, smoking status, diabetic status, and cholesterol levels as predictors [11]. Recalibration was performed by calculating the baseline survival and mean risk factor values from a Bavarian population–based cohort study (Cooperative Health Research in the Region Augsburg, KORA-S4) [15]. All 3 scores predict the 10-year CVD risk.

The FRS defines CVD as coronary artery disease (CAD; coronary death, myocardial infarction, coronary insufficiency, and angina), cerebrovascular events (ischemic stroke, hemorrhagic stroke, and transient ischemic attack), peripheral arterial disease (PAD; intermittent claudication), and heart failure [11]. SCORE2 and SCORE2-OP define CVD as the composite of cardiovascular mortality (including death because of CAD, heart failure, stroke, and sudden death), nonfatal myocardial infarction, and nonfatal stroke [13,14]. In contrast to the FRS and SCORE2-OP model, the SCORE2 model does not consider the DM as a predictor. Therefore, the FRS is used for all diabetics <70 years of age. The models, their predicted outcomes, and the parameters used are highlighted in Table 1. If users were not aware of their SBP or cholesterol levels, age- and sex-specific mean values were used instead. These mean values were derived from the Bavarian KORA-FF4 cohort.

**Table 1:** Risk scores used in the HerzFit risk calculator^a^.

	Origin	Age range used in HerzFit (years)	Predictors	Predicted outcome	Special points
Framingham Risk Score^b^ [11]	United States	30-39^c^	DM^d^ Smoking SBP^e^+BP^f^ treatment^g^ TC^h^+HDL-C^i^	10-year risk of CVD^j^	Recalibrated to the Bavarian population [15].
SCORE2^k^ [13]	Europe	40-69	Smoking SBP TC+HDL-C	10-year risk of CVD	Version for moderate-risk region is used.
SCORE2-Older Persons [14]	Europe	70-89	DM Smoking SBP TC+HDL-C	10-year risk of CVD	Version for moderate-risk region is used.

^a^All 3 risk scores base their risk estimation on sex and age.

^b^Framingham Risk Score for 10-year risk of cardiovascular disease.

^c^For individuals with diabetes, the Framingham Risk Score ranged from 30 to 69 years.

^d^DM: diabetes mellitus.

^e^SBP: systolic blood pressure.

^f^BP: blood pressure.

^g^Denotes antihypertensive treatment.

^h^TC: total cholesterol.

^i^HDL-C: high-density lipoprotein cholesterol.

^j^CVD: cardiovascular disease.

^k^SCORE2: Systematic Coronary Risk Evaluation 2.

For individuals who already had a CVD event, the 10-year risk of recurrent events was estimated using the Secondary Manifestations Of Arterial Disease (SMART) risk score. Established CVD is defined as the presence of CAD (angina pectoris, myocardial infarction, or coronary revascularization), cerebrovascular disease (transient ischemic attack, cerebral infarction, amaurosis fugax or retinal infarction, or a history of carotid surgery), PAD (symptomatic and documented obstruction of the distal arteries of the leg or angioplasty, bypass, or amputation of the leg), or abdominal aortic aneurysm. Recurrent events were defined as the composite of cardiovascular death, ischemic or hemorrhagic stroke, and myocardial infarction. The SMART risk score was developed on the basis of the SMART study in the Netherlands. The model is based on sex, age, as well as the underlying type of CVD (CAD, cerebrovascular disease, PAD, or aortic aneurysm) and the years since the first event. Smoking status, DM, SBP, total cholesterol (TC) and high-density lipoprotein cholesterol (HDL-C) levels, glomerular filtration rate, and high-sensitivity C-reactive protein level were used as predictors [16].

### CVD Risk Communication

In primary prevention, CVD risk is reported as an individual’s heart age. The heart age is calculated by comparing the individual risk with average risk levels at a given age and thus matching the age of a person with average risk at that age in the population (Table 2). In secondary prevention, the risk calculator provides an individual absolute risk of recurrence instead of the heart age. The same applies to individuals with excessive risk factor levels in primary prevention. In these cases, the heart age of a person would otherwise drastically exceed the biological age (eg, heart age >100 years).

**Table 2:** Average risk factor levels used for heart age estimation. Heart age was estimated by comparing the individual risk with average risk levels. Heart age matches the age of a person with the same predicted risk but with average risk factor levels.

Risk factors	Primary prevention
Smoking	Nonsmoker
Diabetes mellitus	Nondiabetic
Systolic blood pressure	130 mm Hg
Blood pressure treatment^a^	No
Total cholesterol	200 mg/dL
High-density lipoprotein cholesterol	50 mg/dL

^a^Denotes antihypertensive treatment.

Individual risk was compared with the mean risk and optimal risk in both primary and secondary prevention. Mean risk was derived from the Bavarian KORA-FF4 (for individuals without CVD) and F4 (for individuals with CVD) cohorts. The optimal risk was obtained by estimating someone’s risk at the same age but with optimal risk factor levels (Table 3). In addition to traditional risk factors (eg, SBP, DM, and cholesterol levels), the HerzFit risk calculator asks users about their habits related to diet, exercise, and stress management. As these factors are not included in the risk models used for prediction, they do not directly affect the results. However, they are used for individualized recommendations within the app.

**Table 3:** Optimal risk factor levels used for comparison of individual and optimal risk. The HerzFit calculator compares individual risks with the optimal risk level. These parameters were used to obtain the optimal risk. In secondary prevention, the optimal risk is estimated for individual-specific underlying cardiovascular disease and the individual time span since the first event.

	Primary prevention	Secondary prevention
Smoking	Nonsmoker	Nonsmoker
Diabetes mellitus	Nondiabetic	Nondiabetic
Systolic blood pressure	120 mm Hg	120 mm Hg
Blood pressure treatment^a^	No	—^b^
Total cholesterol	150 mg/dL	110 mg/dL
High-density lipoprotein cholesterol	50 mg/dL	50 mg/dL
Glomerular filtration rate	—	80 mL/min
C-reactive protein	—	0.1 mg/dL

^a^Denotes antihypertensive treatment.

^b^Not applicable; information not needed for risk estimation.

### Automatic Updates and Trends

The risk calculator was integrated into the mHealth app HerzFit, which allows people to monitor their vital and laboratory parameters. Every time novel risk factor levels are implemented (eg, when a new SBP value is transferred to the HerzFit app), the risk assessment automatically updates itself. This enables users to see how changes in risk factor levels can lead to improvements in their cardiovascular risk, without repeating the assessment every time. Obtaining a more accurate risk prediction, not a single but the mean value of the last 7 measurements of SBP was used for risk estimation. The HerzFit app is available free of charge for both the iOS and Android platforms in Germany, Austria, and Switzerland.

### Data Security

All data (that are or were) entered into the HerzFit risk calculator, and the HerzFit app remained locally on the users’ smartphones. The risk analysis was performed directly on the users’ devices. This privacy by design concept ensures data security for users; however, it limits the statistical evaluation of its use. The strategy was communicated and aligned with the Bavarian Data Protection Commissioner (Landesdatenschutzbeauftragter).

### Ethical Considerations

In this study, the download numbers of HerzFit were extracted from the respective iOS and Android developer accounts. These data cannot be associated with specific individuals and are therefore considered anonymized and fall outside the scope of the European Data Protection Regulation [17]. No institutional review board approval was obtained for our retrospective analysis of these anonymized data [18].

## Results

### Risk Assessment

**Figure 1:**
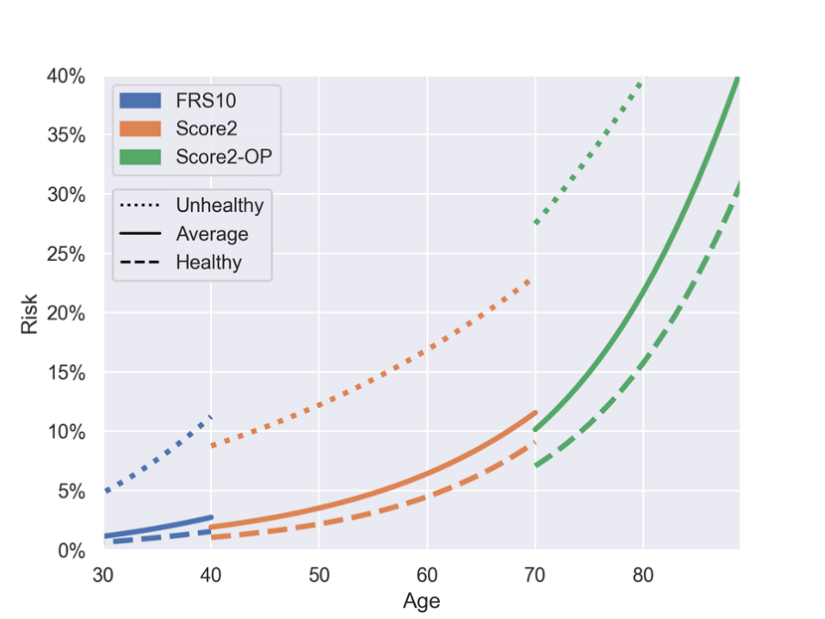
Correlation of age and 10-year risk of cardiovascular disease for different personas. This figure demonstrates the correlation between age and 10-year risk of cardiovascular disease for unhealthy, average, and healthy personas for Framingham Risk Score (FRS), Systematic Coronary Risk Evaluation 2 (SCORE2), and SCORE2–Older Persons (SCORE2-OP) in their respective age ranges used in the HerzFit risk calculator. Healthy persona: male, nonsmoker, nondiabetic, systolic blood pressure (SBP) 125 mm Hg, no blood pressure (BP) treatment, total cholesterol (TC) 170 mg/dL, and high-density lipoprotein cholesterol (HDL-C) 70 mg/dL. Average persona: male, nonsmoker, nondiabetic, SBP 130 mm Hg, no BP treatment, TC 200 mg/dL, and HDL-C 50 mg/dL. Unhealthy persona: male, smoker, nondiabetic, SBP 155 mm Hg, no BP treatment, TC 250 mg/dL, and HDL-C 40 mg/dL.

**Figure 2:**
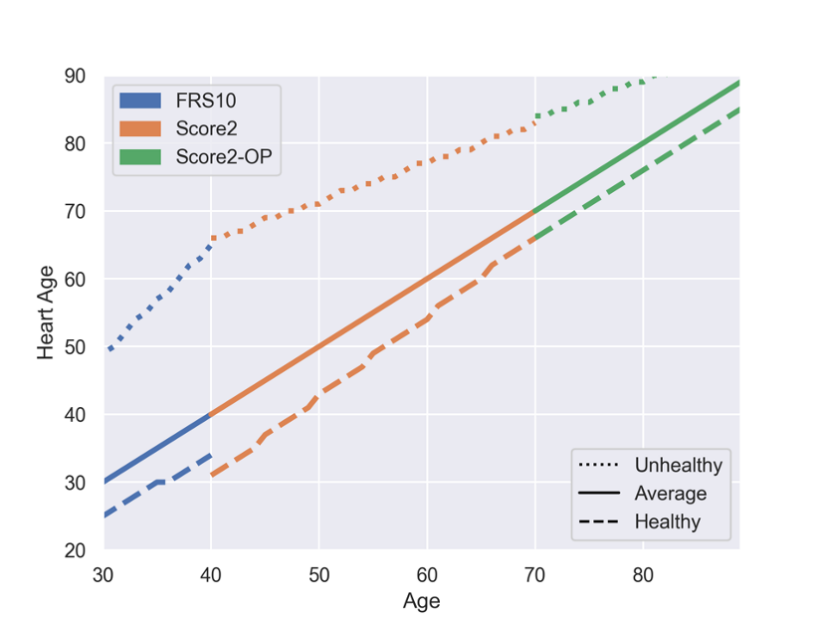
Correlation of age and heart age in the HerzFit risk calculator. This figure demonstrates the correlation of real age and heart age for unhealthy, average, and healthy individual personas for Framingham Risk Score (FRS), Systematic Coronary Risk Evaluation 2 (SCORE2), and SCORE2–Older Persons (SCORE2-OP) in their respective age ranges used in the HerzFit risk calculator. Healthy persona: male, nonsmoker, nondiabetic, systolic blood pressure (SBP) 125 mm Hg, no blood pressure (BP) treatment, total cholesterol (TC) 170 mg/dL, and high-density lipoprotein cholesterol (HDL-C) 70 mg/dL. Average persona: male, nonsmoker, nondiabetic, SBP 130 mm Hg, no BP treatment, TC 200 mg/dL, and HDL-C 50 mg/dL. Unhealthy persona: male, smoker, nondiabetic, SBP 155 mm Hg, no BP treatment, TC 250 mg/dL, and HDL-C 40 mg/dL.

For primary prevention, neither FRS (applicable to individuals aged ≥30 years) nor SCORE2 models (SCORE2 applicable to individuals aged 40-69 years; SCORE2-OP applicable to individuals aged ≥70 years) can be used for the complete range of age and spectrum of risk factors. As we had to integrate these instruments into a single calculator, we had to harmonize the transition zones between the respective risk scores. Moreover, because of differences in their underlying study populations, the absolute risks derived from the FRS differed from the absolute risks derived from the SCORE2 model. Despite being recalibrated to the German population, the FRS still tends to calculate higher absolute risks compared with the SCORE2 model. This difference in absolute risk was more prominent in higher-risk individuals. Presenting a heart age instead of absolute risks is intended to mitigate this problem and ensure smoother transitions between the FRS and the SCORE2 model. Despite the similarities in the underlying study populations and mathematical calculations, differences in the predicted absolute risks were also evident in the transition from SCORE2 to SCORE2-OP. Similar to the transition between the FRS and SCORE2, the differences were more pronounced in the high-risk groups. Using a heart age instead of absolute risks helped to overcome these differences and ensured a smooth transition between the scores. To illustrate the differences in absolute risk and heart age for individuals with different risk profiles, Figures 1 and 2 and Table 4 illustrate the transition zones between the different scores for 3 example personas: 1 unhealthy persona (smoker, SBP 155 mm Hg, TC 250 mg/dL, and HDL-C 40 mg/dL), 1 average persona (nonsmoker, SBP 130 mm Hg, TC 200 mg/dL, and HDL-C 50 mg/dL), and 1 healthy persona (nonsmoker, SBP 125 mm Hg, TC 170 mg/dL, and HDL-C 70 mg/dL).

**Table 4:** Risk score transition—differences in absolute risk and heart age^a^.

Biological age (years)	Risk score	Healthy persona^b^		Average persona^c^		Unhealthy persona^d^	
		Absolute 10-year risk (%)	Heart age (years)	Absolute 10-year risk (%)	Heart age (years)	Absolute 10-year risk (%)	Heart age (years)
37	FRS^e^	1.23	31	2.17	37	8.96	60
38	FRS	1.33	32	2.35	38	9.68	62
39	FRS	1.44	33	2.54	39	10.44	63
40	SCORE2^f^	1.06	31	1.92	40	8.77	66
41	SCORE2	1.14	32	2.04	41	9.07	66
42	SCORE2	1.22	33	2.17	42	9.37	67
67	SCORE2	7.38	63	9.72	67	21.02	81
68	SCORE2	7.91	64	10.30	68	21.68	82
69	SCORE2	8.49	65	10.92	69	22.36	82
70	SCORE2-OP^g^	7.07	66	10.15	70	27.49	84
71	SCORE2-OP	7.68	67	10.98	71	28.56	84
72	SCORE2-OP	8.33	68	11.87	72	29.67	85

^a^For primary prevention, Framingham Risk Score, Systematic Coronary Risk Evaluation 2 (SCORE2), and SCORE2 in Older Persons were used for risk estimation. Differences in absolute risk estimates were apparent in the transition zones between scores, particularly in high-risk individuals. Estimating the heart age instead of the absolute risk numbers helped smooth the transitions between scores.

^b^Healthy persona: male, nonsmoker, nondiabetic, systolic blood pressure 125 mm Hg, no blood pressure treatment, total cholesterol 170 mg/dL, high-density lipoprotein cholesterol 70 mg/dL.

^c^Average persona: male, nonsmoker, nondiabetic, systolic blood pressure 130 mm Hg, no blood pressure treatment, total cholesterol 200 mg/dL, high-density lipoprotein cholesterol 50 mg/dL.

^d^Unhealthy persona**:** male, smoker, nondiabetic, systolic blood pressure 155 mm Hg, no blood pressure treatment, total cholesterol 250 mg/dL, high-density lipoprotein cholesterol 40 mg/dL.

^e^Framingham Risk Score.

^f^SCORE2: Systematic Coronary Risk Evaluation 2.

^g^SCORE2-OP: Systematic Coronary Risk Evaluation 2–Older Persons.

### Risk Communication

Before users are able to assess their risk, the concept and limitations of risk estimation are explained to the user. It is pointed out that the risk calculator can only estimate but not exactly predict the cardiovascular risk.

**Figure 3:**
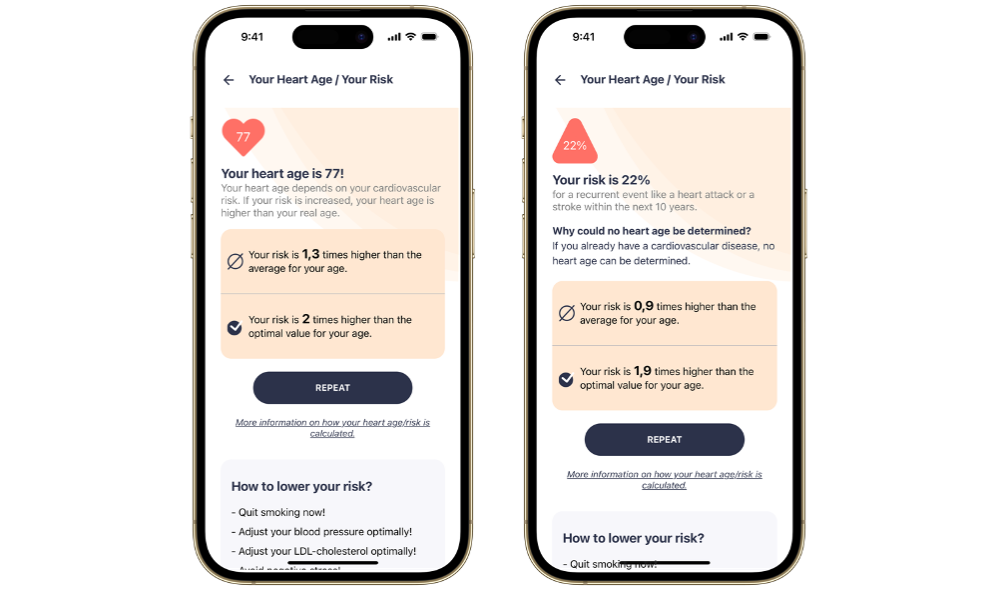
Presentation of heart age and risk in primary and secondary prevention. For individuals without cardiovascular disease, the HerzFit calculator estimates an individual’s heart age. The 10-year risk of recurrent events was estimated for people with established cardiovascular disease. In addition, the individual risk is compared with the mean and optimal risk. The screenshots were translated from German.

**Figure 4:**
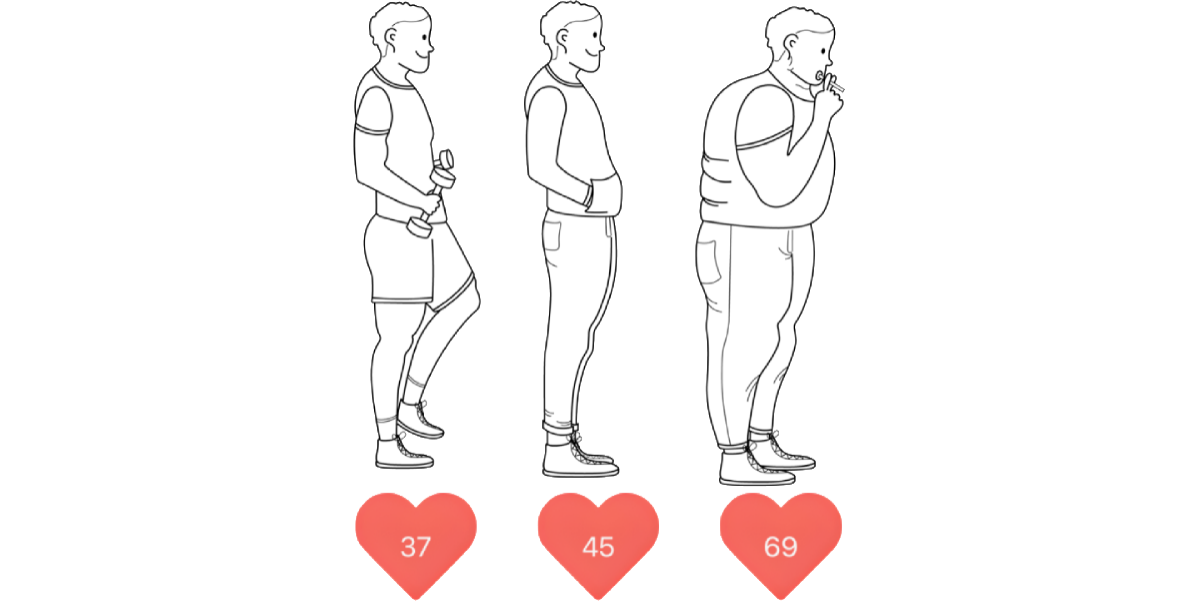
The effect of different risk profiles on heart age. This figure shows the effects of cardiovascular risk factors on heart age. Although a healthy man aged 45 years has a heart age of 37 years, and an average man of the same age has a heart age of 45 years, an unhealthy man of the same age can already have a heart age of 69 years. Healthy persona: nonsmoker, nondiabetic, systolic blood pressure (SBP) 125 mm Hg, no blood pressure (BP) treatment, total cholesterol (TC) 170 mg/dL, and high-density lipoprotein cholesterol (HDL-C) 70 mg/dL. Average persona: nonsmoker, nondiabetic, SBP 130 mm Hg, no BP treatment, TC 200 mg/dL, and HDL-C 50 mg/dL. Unhealthy persona: smoker, nondiabetic, SBP 155 mm Hg, no BP treatment, TC 250 mg/dL, and HDL-C 40 mg/dL.

After entering the required parameters, the heart age (in primary prevention) or the risk of recurrence (in secondary prevention) and the comparison to the mean and optimal risk are depicted (Figure 3). If risk factor levels change (eg, someone stops smoking or takes better care of blood pressure [BP] levels), individual risk and thereby heart age automatically adapt. Figure 4 uses the 3 example personas described above (1 healthy, 1 average, and 1 unhealthy) to illustrate how different risk profiles affect the heart age. For the average persona, who does not smoke, has no diabetes, and has normal BP and cholesterol levels, biological age and heart age match. The healthy persona, who has even better BP and cholesterol levels than the population average, has a younger heart age than the biological one. This persona shows a potential gain in life years. The unhealthy persona, on the other hand, who smokes and has elevated BP and cholesterol levels, is given an increased heart age. This persona is made aware of the possible loss of life years.

### Raising Awareness

**Figure 5:**
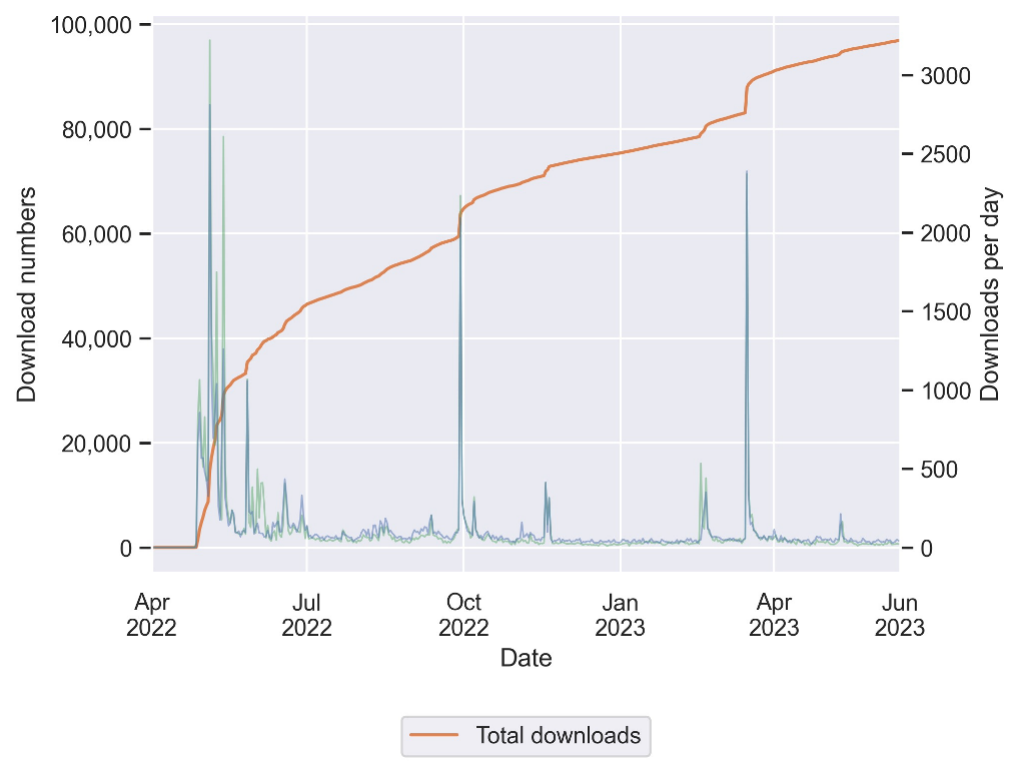
HerzFit downloads from April 2022 to June 2023. Since its release in April 2022, the HerzFit app and risk calculator have been downloaded more than 96,000 times. The orange curve represents the total number of downloads. The green and blue curves mark the downloads per day on iOS and Android devices, respectively. In addition to a continuous increase in the total download numbers, repeated peaks in daily downloads were evident. This finding could be correlated with previous marketing campaigns and events.

Within the first 14 months, the HerzFit app and its risk calculator were downloaded more than 96,000 times. In addition to a steady increase in the number of users, several download peaks were evident (Figure 5). The greatest increase in the number of users occurred in the months following the release in April 2022. Before and immediately after the release, many marketing activities were carried out by the State of Bavaria, the German Heart Foundation, the Digitized Medicine in Bavaria (DigiMed Bavaria) Consortium, a cooperating health insurance company, and a health magazine. In addition, the HerzFit app and its risk calculator were promoted as part of a large-scale heart health campaign (Hand aufs Herz Kampagne) funded by the Bavarian State Ministry of Health and Care. After the first 3 months, a slower but steady increase in the number of users was observed. The next major download peak was evident in the fall of 2022, when the German Heart Foundation presented the risk calculator at several events for the World Heart Day. In addition, a scientific symposium organized by the DigiMed Bavaria Consortium took place, which subsequently led to broader attention for the risk calculator in the media. Finally, a third major peak in downloads could be observed in the spring of 2023 when the German Heart Foundation and a health magazine once again promoted the HerzFit risk calculator.

## Discussion

### Principal Findings

This study indicates the feasibility of and public demand for a calculator that indicates the risk of CVD. The main findings of our study are as follows: offering a calculator that covers a broad range of ages for both primary and secondary prevention required use of multiple instruments. For individuals covered by >1 established risk assessment instrument, estimates may differ significantly, which implies the need for harmonization to cover a broad range of age and risk profiles in a single tool. Providing plausible estimates that can be interpreted by laypersons is essential for risk communication. Depending on public campaigns that create awareness, the public shows great interest in a CVD self-assessment tool.

A novel aspect of this study is the integration of multiple primary and secondary preventive risk scores into a single instrument for laypersons. Previous self-assessment tools were mostly based on a single primary preventive score such as the FRS or the QRESEARCH cardiovascular risk algorithm model [7,19]. As a result, certain groups of age and risk profiles, as well as individuals with preexisting CVD, were excluded from the risk estimation. More complex risk assessment instruments, such as the web-based U-Prevent tool or the European Society of Cardiology CVD Risk Calculation App, provide risk assessment for people in both primary and secondary prevention. However, they were not developed as a self-assessment tool for laypersons but rather as a clinical decision support platform for physicians [20,21]. Given these limitations, we integrated several primary and secondary preventive scores into a single instrument for laypersons. Following the current guideline recommendations, SCORE2 models and the SMART risk score form the basis of the HerzFit calculator [1,13,14]. A recalibrated version of the FRS complements our tool by providing risk estimation for younger individuals and those with preexisting diabetes [15,22]. Designed as a self-assessment tool for laypersons, the HerzFit calculator automatically uses the appropriate risk model based on the individual information on risk factors and disease history. Similar to previous self-assessment tools, mean values are automatically provided to ensure risk estimation when individuals are unaware of their laboratory values [7]. Combining different primary and secondary preventive risk models, the HerzFit calculator covers a broad range of age and risk profiles as well as patients with previous cardiovascular events [23]. This approach was encouraged by our cooperating patient organization, the German Heart Foundation, as a large proportion of their members already have CVD. It was also supported by the Bavarian State Ministry of Health and Care, which needed a widely applicable risk assessment and awareness tool for a large-scale heart health campaign.

The integration of multiple scores with different underlying study populations requires recalibration and harmonization to obtain consistent and plausible estimates. In primary prevention, calculating the heart age instead of absolute risks ensured the harmonization of results. Presenting a heart age also promised to facilitate risk communication, as percentage risk formats have been shown to be difficult for laypersons to understand [24]. Several randomized trials have compared the effects of communicating the heart age instead of absolute risk percentages. The study by Soureti et al [24] showed that heart age had greater emotional impact in individuals at higher cardiovascular risk. The study by Lopez-Gonzalez et al [25] found that a heart age tool led to greater reduction in CVD risk compared with conventional 10-year risk percentages. However, the literature also indicates that high-risk individuals were less accepting of their heart age results and perceived them as less credible [26,27]. Given these limitations, an additional comparison of expected to mean and optimal risk levels using risk ratios was implemented in the HerzFit calculator. Risk ratios and peer group risk estimates have been shown to increase risk perception and intention to adhere to a healthier lifestyle [28]. In contrast to other apps, we refrained from showing absolute 10-year risks in addition to the heart age [29]. Especially younger users, who may already have an increased relative risk and heart age because of modifiable risk factors, could otherwise be misled by low absolute risk numbers. A graphical representation of the estimated risk as in other CVD risk estimation apps has not yet been implemented in the HerzFit risk calculator [23,29].

Another specialty of the HerzFit risk calculator is how it is delivered. Previous self-assessment tools were mostly web based or developed as stand-alone apps for risk assessment [7,19,20,23,29]. In contrast to these delivery methods, the HerzFit risk calculator was developed as a smartphone tool and integrated into an mHealth app. This app not only provides respective background information but also encourages its users to better manage their health based on the individual risk assessment. In this way, the HerzFit calculator could not only help identify individuals at risk but also help them optimize their cardiovascular profile.

Finally, our study demonstrated great public interest in the German-speaking population for a CVD self-assessment tool. This result is consistent with those of previous studies conducted in the United States and the United Kingdom [7,19]. With up to 10,000 downloads per month, public information campaigns have had a significant impact on download numbers. This also supports previous studies reporting increased disease and risk factor awareness following public information campaigns [19,30-32].

### Strengths and Limitations

This study has several strengths. To the best of our knowledge, the HerzFit calculator is the first smartphone-based tool to offer guideline-based CVD risk estimation for laypersons in German-speaking countries. The integration of 4 internationally recommended scores allows risk estimation for individuals aged 30 to 90 years with or without CVD. Recalibration and harmonization ensure plausible estimates based on individual risk profiles. Presenting a heart age and comparing the expected to mean and optimal risk levels promise better risk comprehension among users. Finally, the calculator is implemented into an mHealth app that provides the respective background information and is delivered free of charge on both the iOS and Android platforms.

The following limitations of this study must be addressed. First, due to the data protection regulations, there has been no statistical evaluation of the user profile of the HerzFit calculator. It remains unclear whether HerzFit reaches at-risk individuals and whether it has an impact on their behavior. However, recent studies have revealed a great interest in CVD self-assessment tools among all risk groups and showed considerable improvement in risk factor control when heart age was communicated [19,24,25]. Developed with the functionality to collect scientific data, the HerzFit app may soon provide further evidence in this regard. With user consent, anonymized data on individual risk factors, comorbidities, and estimated risks shall be analyzed in the future. The results could even be used to guide future campaigns to create awareness for the instrument. Second, risk scoring in general faces limitations when it comes to individuals with extreme risk factor levels (eg, familial hypercholesterolemia) [33]. Conventional risk scoring tools such as ours might underestimate the risk of these persons. This also emphasizes that the HerzFit calculator is not intended to replace a professional health care consultation. Nevertheless, self-assessment tools can be seen as a way to provide information, direct people toward traditional preventive measures, and empower them in their health management. Finally, the HerzFit calculator is only available in German language so far, which could lower user rates in non–native-speaking groups.

### Conclusions

The HerzFit calculator provides a guideline-based CVD risk assessment for the general population in Germany, Austria, and Switzerland. With more than 96,000 downloads within the first 14 months, the great public demand for such a self-assessment tool was demonstrated. Public information campaigns were largely responsible for creating awareness about the instrument.

## Abbreviations

BP: blood pressure
CAD: coronary artery disease
CVD: cardiovascular disease
DM: diabetes mellitus
FRS: Framingham Risk Score
HDL-C: high-density lipoprotein cholesterol
mHealth: mobile health
PAD: peripheral arterial disease
SBP: systolic blood pressure
SCORE2: Systematic Coronary Risk Evaluation 2
SCORE2-OP: Systematic Coronary Risk Evaluation 2–Older Persons
SMART: Secondary Manifestations Of Arterial Disease
TC: total cholesterol

## Data Availability

The data that support the findings of this study are available from the corresponding author upon reasonable request.
